# Single Spheroid Metabolomics: Optimizing Sample Preparation of Three-Dimensional Multicellular Tumor Spheroids

**DOI:** 10.3390/metabo9120304

**Published:** 2019-12-14

**Authors:** Mate Rusz, Evelyn Rampler, Bernhard K. Keppler, Michael A. Jakupec, Gunda Koellensperger

**Affiliations:** 1 Institute of Inorganic Chemistry, University of Vienna, Währinger Str. 42, 1090 Vienna, Austria; mate.rusz@univie.ac.at (M.R.); bernhard.keppler@univie.ac.at (B.K.K.); michael.jakupec@univie.ac.at (M.A.J.); 2 Institute of Analytical Chemistry, University of Vienna, Währinger Str. 38, 1090 Vienna, Austria; evelyn.rampler@univie.ac.at; 3 Vienna Metabolomics Center (VIME), University of Vienna, Althanstraße 14, 1090 Vienna, Austria; 4 Research Network Chemistry Meets Microbiology, Althanstraße 14, 1090 Vienna, Austria

**Keywords:** multicellular tumor spheroids, metabolomics, metallodrugs, oxaliplatin, KP1339, method development, LC-MS, IT-139

## Abstract

Tumor spheroids are important model systems due to the capability of capturing in vivo tumor complexity. In this work, the experimental design of metabolomics workflows using three-dimensional multicellular tumor spheroid (3D MTS) models is addressed. Non-scaffold based cultures of the HCT116 colon carcinoma cell line delivered highly reproducible MTSs with regard to size and other key parameters (such as protein content and fraction of viable cells) as a prerequisite. Carefully optimizing the multiple steps of sample preparation, the developed procedure enabled us to probe the metabolome of single MTSs (diameter range 790 ± 22 µm) in a highly repeatable manner at a considerable throughput. The final protocol consisted of rapid washing of the spheroids on the cultivation plate, followed by cold methanol extraction. ^13^C enriched internal standards, added upon extraction, were key to obtaining the excellent analytical figures of merit. Targeted metabolomics provided absolute concentrations with average biological repeatabilities of <20% probing MTSs individually. In a proof of principle study, MTSs were exposed to two metal-based anticancer drugs, oxaliplatin and the investigational anticancer drug KP1339 (sodium *trans*-[tetrachloridobis(1*H*-indazole)ruthenate(III)]), which exhibit distinctly different modes of action. This difference could be recapitulated in individual metabolic shifts observed from replicate single MTSs. Therefore, biological variation among single spheroids can be assessed using the presented analytical strategy, applicable for in-depth anticancer drug metabolite profiling.

## 1. Introduction

Three-dimensional multicellular tumor spheroids (3D MTSs) emerged as essential tools in cancer research with the aim of increasing the efficiency of oncologic drug development. Indeed, MTSs provide a cancer model of intermediate complexity, not replacing animal models entirely, but constituting a substantial improvement compared to two-dimensional (2D) monolayer cell cultures [1,2]. 3D MTSs grown from established cancer cell lines resemble more closely early-stage avascular tumors than 2D monolayer cell cultures in many aspects. For example, nutrient and oxygen concentration gradients as present in tumors are established in 3D MTS models, which in turn results in a concentration of proliferating cancer cells in the outer layers of the MTS, while in the inner zone, deprived of nutrients and oxygen, necrotic cells accumulate (depending on the MTS size). In addition, viable quiescent cells are found in a transition zone between the necrotic core and outer layer of proliferating cells [3]. This inhomogeneity is an important feature of in vivo tumors, which 2D monolayer cell cultures fail to recapitulate as they predominantly contain normoxic, actively proliferating cells [2,4]. Many anticancer drugs exert their cytotoxicity against proliferating cells, with quiescent cells evading the treatment [5]. Thus, a model system such as 3D MTSs, which involve quiescent cells, is of utmost importance.

Another key aspect is the fact that the three-dimensionality intrinsically affects the cell morphology (rather round instead of stretched-out on a plastic surface), which relates to cell-to-cell contacts, stimuli exerted by cell surface receptors, and, ultimately, transcription and protein expression levels [6]. Furthermore, reduced oxygen levels and hypoxia lead to the generation of reactive oxygen species (ROS) and hypoxia-inducible factor-1 (HIF-1) stabilization, which is a major transcription factor responsible for metabolic transformation and tumor progression [7,8,9,10].

In the recent past, the combination of cancer models with cutting edge –omics type of analysis showed to be a powerful approach for generating new hypotheses regarding the prediction of drug susceptibility, drug resistance, and mode of action [11,12]. This was accompanied by a reemerging interest in cancer metabolism [13]. Metabolic signatures are accepted as the closest proxy for a phenotype [14,15]. One of the biggest hopes in cancer metabolomics is the discovery of molecular signatures with predictive power in cancer therapy [16]. As a consequence of this renewed interest in metabolism, dedicated workflows were introduced, addressing the needs of preclinical and clinical studies [17,18,19]. Cancer cell model studies required the development and validation of multi-step sample preparation protocols [20]. While today metabolomics experiments with 2D monolayer cell cultures are routine, sample preparation protocols for 3D MTSs are rarely discussed in detail. Up to date, only a few reports on metabolomics in 3D MTS models exist. The studies involved a range of LC-MS-based metabolomics workflows including lipidomics and fluxomics applications [21,22,23,24,25]. Despite showing the power of combining advanced models such as 3D MTSs and metabolomics, the validation of sample preparation was not addressed comprehensively. Even a detailed description of the experimental design (e.g., whether MTS samples were pooled for the analysis or how gels established in 3D culture were removed upon metabolome extraction) was lacking.

In this work, we focus on the experimental design enabling metabolomics in 3D MTSs, using non-scaffold based cell cultures grown on ultra-low attachment plates from cell suspensions. This approach avoids 3D scaffolds or gels for obtaining a three-dimensional structure, which facilitates metabolomics measurements. More specifically, sample preparation protocols are developed with the goal to provide a validated procedure capable of probing single MTSs; at the same time not compromising on analytical throughput and, thus, the number of biological replicates. The validity of the established preclinical tool-set was shown for the example of metal-based anticancer drug development. Metal-based drugs are a prime example since a clear cut mechanism remains to be elucidated despite extensive clinical use and fundamental research [26,27]. In fact, how the drugs exert their cytotoxicity differs even for the three clinically approved platinum(II) drugs [28,29,30]. Although massive research efforts resulted in a plethora of promising candidate drugs, the failure rate upon translation into clinics was/is extremely high for metal-based anticancer drugs [31]. Discovering potential metabolic pertubations specific for drug exposure, by measuring molecular signatures in advanced 3D MTS models, bear the potential of accelerating discoveries with regard to the mode of action and susceptibility towards the drug. In this work, a 3D human colon cancer model was studied. Metabolic shifts, as exerted by the clinically established oxaliplatin and KP1339, a promising candidate drug, were investigated.

## 2. Results

### 2.1. Establishment and Validation of Sample Preparation Procedures Suitable for Probing the Metabolome of Spheroids

#### 2.1.1. Determining the Minimal Number of MTS Required for Metabolomics Experiment

A uniform size distribution of MTSs was obtained by using the colon cancer model HCT116; seeding of 3 × 10^3^ cells and cultivation for 4 days following the suspension-based procedure described in the experimental section. The average resulting spheroid diameter was 560 ± 30 µm (*n* = 56). Suspension-based 3D cultivation enabled a straightforward establishment of metabolomics workflows otherwise hampered by tedious washing procedures in hydrogel and other scaffold-based techniques. The investigated size range amounted to 9.9 × 10^3^ ± 3.9 × 10^3^ cells with 81% ± 5% viable cells (Table S1, “cellNumbers” sheet). The estimation was based on disaggregating the MTS with a recombinant enzyme reagent, staining with a dye, and subsequent measurement with a flow cytometer to determine cell counts and viability in the final suspension.

In order to exclude poor extraction efficiencies for MTSs in this work, metabolomics experiments resorted to boiling ethanol extractions, implementing the yeast-derived fully ^13^C labeled (U^13^C) internal standard. More specifically, extractions were carried out on single spheroids (three biological replicates, *N* = 3) and pooled spheroid samples, i.e., pools of 5 (*N* = 3), 10 (*N* = 3) and 15 spheroids (*N* = 2), respectively. The applied boiling ethanol protocol is the established gold standard in yeast metabolomics, offering nearly 100% extraction efficiency and recovery for a large panel of primary metabolites [32,33]. In cancer cell monolayer cultures, less tedious cold extraction protocols are established, which demand thorough validation when applied to MTS investigations [20,34].

**Figure A2:**
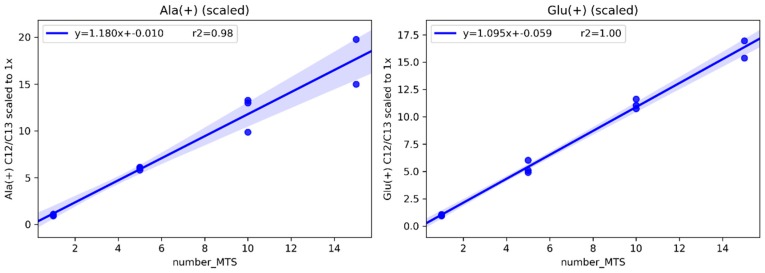
Scaled relative responses (^12^C/^13^C) of alanine and glutamate as selected examples from a single (*N* = 3), 5 (*N* = 3), 10 (*N* = 3), 15 (*N* = 2) multicellular tumor spheroids: relative responses were scaled to the mean relative response in single multicellular tumor spheroids (*N* = 3). Sample preparation applied boiling ethanol extraction with U^13^C internal standardization and LC high-resolution mass spectrometry. Alanine and glutamate are shown from the investigated compounds.

**Figure A3:**
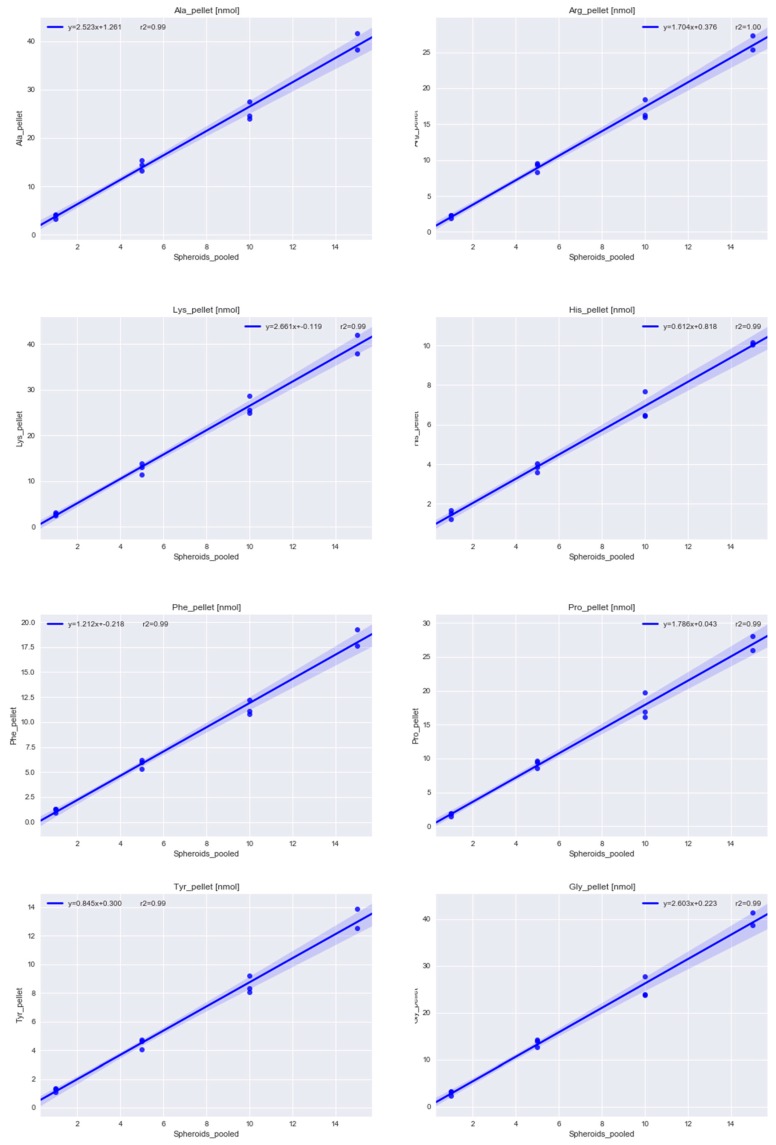
Absolute amounts in nmol of amino acids after acidic hydrolysis of the pellet containing 1 (*N* = 3), 5 (*N* = 3), 10 (*N* = 3) and 15 (*N* = 2) multicellular tumor spheroids. The pellets are resulting from the high molecular fraction of the boiling ethanol extraction and centrifugation. Amino acid amounts from the proteins show a strong linear correlation with the number of spheroids in the sample.

Targeted metabolomics measurements were performed implementing reversed-phase chromatography coupled to tandem mass spectrometry using a 100%-wettable column providing enhanced separation for branched amino acids and organic acids [35]. Metabolite abundances relative to the isotopically enriched internal standard, i.e., relative response ratios, were addressed in single MTS extractions versus pooled extractions. The validation considered 29 metabolites (amino acids, organic acids, nucleotides, nucleosides). Single spheroid extractions resulted on average in repeatabilities of 28%, while pooled samples showed repeatabilites of 17% and 32% for 5 and 10 MTSs pooled, respectively (Table S1, “metabolitesExtract” sheet). In order to evaluate whether the number of pooled MTSs correlated with cell number and protein content, the cell pellets remaining upon boiling ethanol were submitted to acidic hydrolysis. Absolute amounts of 8 amino acids (alanine, arginine, glycine, histidine, lysine, phenylalanine, proline, tyrosine) were determined. The selected amino acids were quantitatively recovered [36] by the applied sample preparation protocol and were used for traceable protein quantification. The obtained absolute amino acid amounts displayed a strong linear correlation with the number of MTSs (Table S1, “aminoAcids_pellet”, and Figure A3). This linear correlation (coefficient of determination above 0.99) was a prerequisite for further evaluation of metabolome abundances in single versus pooled MTS samples, depending on the assumption of linear correlation between cell number, protein concentration, and the number of spheroids (for a uniformly sized MTS sample set). Upon transforming the linear regressions of metabolite abundances from intracellular cell extracts versus number of spheroids by normalizing the metabolite abundances to average abundance found in single MTS samples, the parameters of the linear regression became comparable: assuming an ideal scenario (as represented by 100% extraction efficiency and recovery regardless of whether pooled or single samples are investigated) for these plots, a slope of 1 is expected and can be observed for the investigated metabolites, the ideal case of slope of 1 is nearly met (Table S1, “regressionNormalized”, Figure A2 and Figure A3). On average, the slope of the normalized regression is 1.11 and the average coefficient of determination is 0.94. Overall, the strong linear correlation of the relative responses with the number of uniform-size spheroids indicates that metabolomics experiments, even from single spheroids, are highly repeatable.

**Figure 1:**
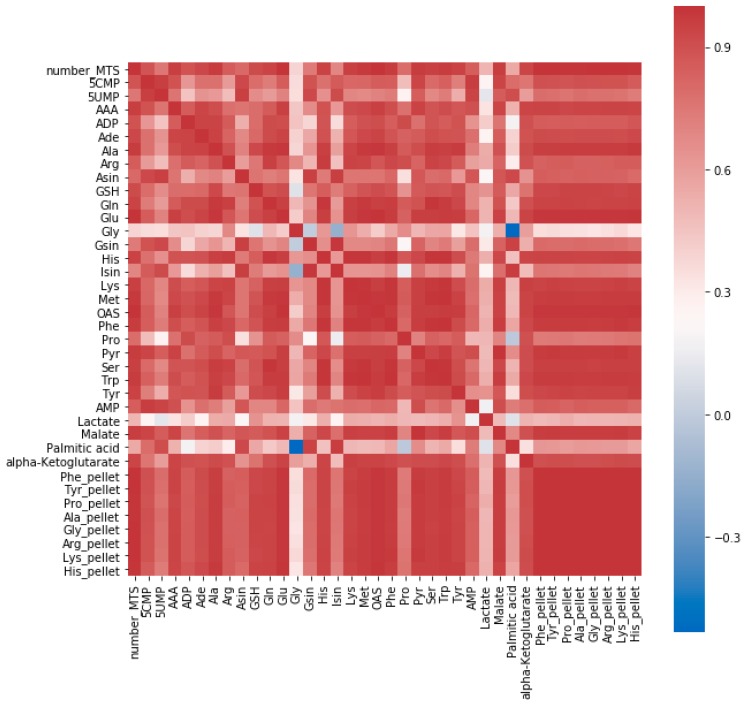
Correlation matrix of number of multicellular tumor spheroids (MTS), relative responses (^12^C/^13^C) of 29 metabolites from boiling ethanolic extracts with ^13^C internal standardization, absolute amounts of 8 amino acids [nmol] measured from hydrolyzed pellet (indicated with the “pellet” suffix) from samples containing 1 (*N* = 3), 5 (*N* = 3), 10 (*N* = 3), and 15 MTS (*N* = 2) from the same population. Most metabolites show a strong correlation with all the amino acids hydrolyzed from the pellet and the number of MTSs.

These findings are captured in a correlation matrix (Figure 1), which reveals that most of the investigated metabolites have a strong positive correlation with the amino acids measured from the corresponding pellet as well as from the number of MTSs they were extracted from.

#### 2.1.2. Speeding Up the Sample Preparation

In the next step, alternative extraction protocols were addressed with the aim of increasing analytical throughput without compromising overall accuracy. The workflow based on boiling ethanol, involved the transfer of MTSs into tubes, three washing steps, and quenching by flash freezing with liquid nitrogen, which was followed by hot extraction. As a drawback, the procedure is rather time-consuming and on top of that, only a very limited number of MTS samples can be prepared in parallel. In fact, during collection and washing, only a limited number of spheroids can be handled at a given time. Only after the handled samples are quenched can be the next MTS collected. This often implies different durations of incubation at uncontrolled temperatures and CO_2_ partial pressure, which might lead to systematic metabolic biases. Thus, it is key to decrease the time until quenching, as only a high degree of synchronization enables reasonable study sizes (number of replicates) and investigations of cells upon multiple metabolic perturbations [20].

In this work, the reduction of cell manipulations was addressed as follows: First, comparative metabolomics experiments compared different strategies regarding the first washing steps. Instead of transferring MTSs to tubes for repeated washing, single-step washing was performed on the cultivation plate directly. (For the sake of clarity the first approach was denoted as “OFF”, while the single washing step on the well-plate was denoted as “ON”.) Second, the extraction procedure was optimized. More specifically, the hot extraction (boiling ethanol, “BE”) was compared to a cold methanol-based extraction (“CM”). In the past, it was shown that the harsh conditions of hot ethanol were not a stringent requirement for monolayer mammalian cell cultures [34], where mild cold extractions proved to be valid strategies.

The already-mentioned studies [18,19,20,21,22] with 3D cultures involving LC-MS-based metabolomics workflows, including lipidomics and fluxomics applications utilized washing of the samples and organic solvents (methanol, acetonitrile) with an aqueous proportion for extraction, mostly in cold state. To the best of our knowledge, a thorough evaluation of 3D cancer cell models is still lacking.

**Figure 2:**
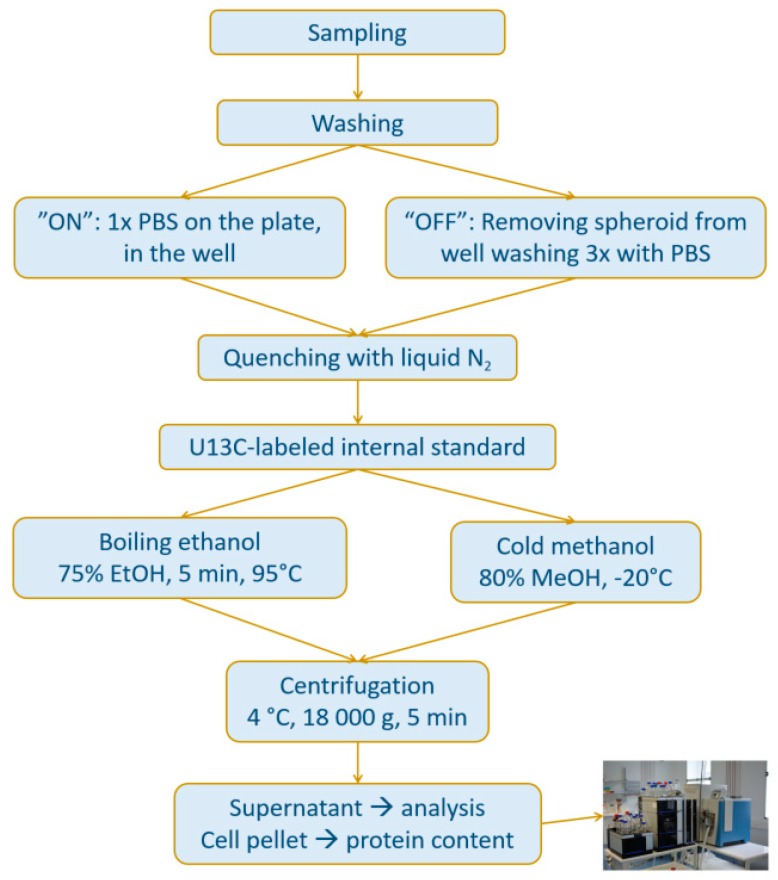
The study design for speeding up the sample preparation. Two different washing procedures (“ON”: washing the multicellular tumor spheroid once with PBS on the 96-well plate vs. “OFF” washing three-times in an Eppendorf-tube after transfer from cultivation well) and two alternative extractions (boiling ethanol vs. cold methanol extraction) were tested on large multicellular tumor spheroids (744 ± 15 µm), which in combination resulted in four sample groups.

Figure 2 depicts the two sample preparation strategies involving washing, quenching, internal standardization using ^13^C internal standards and extraction. The validation experiments were carried out using replicates of single spheroids. The cultivation was designed to produce large spheroids (diameter = 744 ± 15 µm, 4-day long cultivation, and 10^4^ cells seeded) representing a “worst-case scenario” for efficient extraction. Four different sample preparation strategies were compared, namely OFF and ON plate washing, both followed by either BE or CM, respectively, all involving U^13^C internal standardization upon extraction. The extracts were measured as described in [37]. In brief, a hydrophilic interaction liquid chromatography (HILIC) separation at pH 4 was used combined with high-resolution MS. Targeted absolute quantification of 26 metabolites based on external calibration with internal standardization served as validation of the sample preparation.

**Figure 3:**
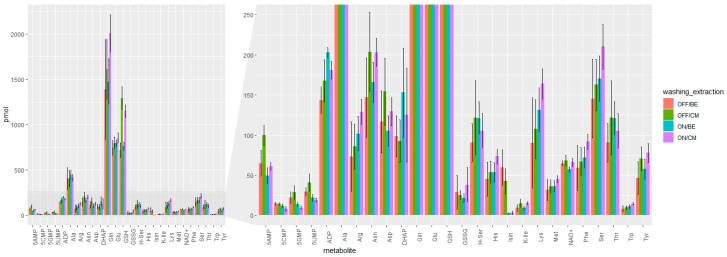
Barplots of the absolute amounts (pmol) of 26 selected metabolites extracted from single multicellular tumor spheroids with four different sample preparation method as well as two washing procedures (“ON”: washing the multicellular tumor spheroid once with PBS on the 96-well plate vs. “OFF” washing three times in an Eppendorf-tube after transfer from the culture plate) and two alternative extractions (boiling ethanol, “BE” vs. cold methanol extraction “CM”). OFF/BE (*N* = 6), ON/BE (*N* = 4), OFF/CM (*N* = 5), ON/CM (*N* = 7) Measurement with liquid chromatography (HILIC separation) high-resolution Orbitrap mass spectrometry. All extractions utilized U^13^C internal standardization. For the abbreviations of compounds, see Appendix A.

In addition to the biological repeatability, the technical repeatability was assessed by injections of a pooled sample over the measurement series (denoted as QC). Figure 3 summarizes the quantitative metabolome data. Overall, it was found that the accelerated workflow with the reduced washing steps was key to improve repeatability, as this resulted in the lowest average relative standard deviations (18% and 19% for ON/BE and ON/CM, respectively. See Table 1.). Moreover, it could be shown that the cold methanol (CM) extraction was comparable to boiling ethanol (BE) in terms of extraction efficiency, as the quantitative values were in good agreement (within their uncertainty).

Thus it can be concluded that the protocol ON/CM offers a fast and convenient fit-for-purpose method to generate 60 biological replicates in parallel. The 60 replicates were treated and washed within a few minutes (depending on the operator) and then were quenched at the same time by flash freezing them with liquid nitrogen. Overall, a 96-well plate accommodates up to 60 spheroid cultivations since wells at the edges are not used to avoid the “edge effect” (artifacts due to inhomogeneity in evaporation rates).

### 2.2. Assessing Metabolic Shifts in Single MTS Exposed to Metal-Based Anticancer Drugs

Finally, the optimized ON/CM workflow was applied in a proof of principle study addressing metabolic perturbations in single MTSs due to exposure of metal-based anticancer drugs. Again human colon cancer cells (HCT116) were selected. The spheroids were grown from 10^4^ cells for 8 days (790 ± 22 µm). Two drugs of distinctly different proposed modes of action were investigated, namely the clinically established oxaliplatin and the investigational anticancer drug sodium *trans*-[tetrachloridobis(1H-indazole)ruthenate(III)] (denoted as KP1339). While oxaliplatin exerts its cytotoxic effects through DNA damage and ribosome biogenesis stress [38], there is growing evidence that KP1339 is not a DNA-damaging agent but its primary mode of action is through endoplasmic reticulum stress [39]. Furthermore, KP1339 shows a prodrug nature, as it is thought to be preferentially reduced in the more reductive milieu of solid tumors to the active Ru(II) form. Finally, while oxaliplatin is considered a bona fide immunogenic cell death inducer [40], KP1339 also exhibits the hallmarks of immunogenic cell death [41,42].

**Figure A4:**
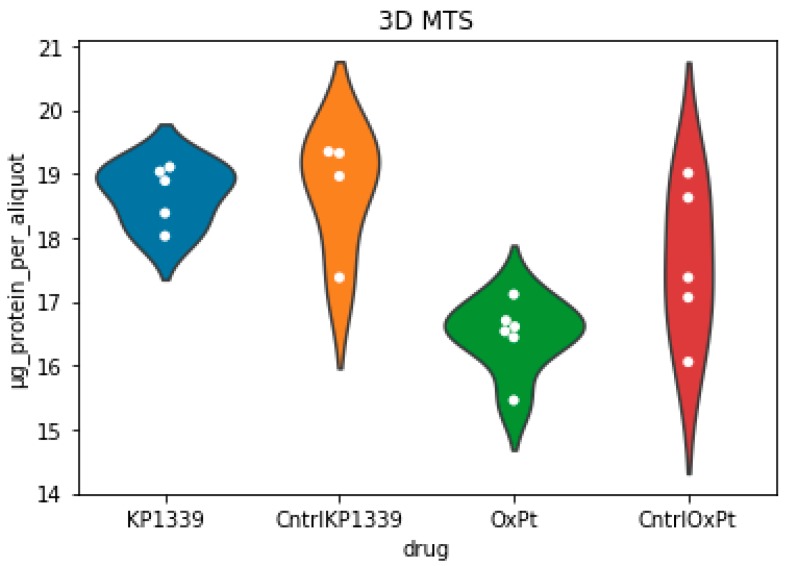
Swarm and violinplot of total protein content [µg] in single 3D MTS cultures (10 × 10^3^ cells seeded, 8 days cultivation diameter 790 ± 22 µm at time of treatment) after 24 h of incubation with 200 µM KP1339, 20 µM Oxaliplatin, growth medium (CntrlOxPt), growth medium with 0.5% DMSO (CntrlKP1339). Protein content is determined by the Thermo micro BCA kit from the high molecular fraction in the pellet.

The choice of incubation time and drug concentration was based on previous studies using monolayer cultures, which showed prominent metabolomic shifts only after 24 h exposure to sub-cytotoxic drug concentrations [37]. Specifically, the applied concentrations were 20 and 200 µM for oxaliplatin and KP1339, respectively. As the dissolution and thus the application of KP1339 involved dimethyl sulfoxide (DMSO), an additional control group resembling the DMSO background in the medium was included. Figure A4 and Table S3 (protein_µg sheet) show the protein concentrations obtained in replicate single MTSs for the different conditions under investigation. The plotted protein content was assessed by measuring the concentration in the remaining cell pellets of the individual MTS samples. As can be readily observed, oxaliplatin treatment resulted in a minor reduction of the overall protein content; otherwise the average mean protein concentration was comparable (within its uncertainty) for all conditions, pointing towards comparable growth rates. On top of that, the data clearly showed that using the average protein content of parallel MTS cultivations would compromise the quality of comparative metabolomics experiments. When aiming at the investigation at single MTS level, it is a requirement to normalize metabolic abundances to the corresponding protein content obtained from the same sample. Finally, the ON/CM sample preparation procedure optimized for single MTS analysis comprised the addition of yeast ^13^C standards. The measurements relied on hydrophilic interaction chromatography—Orbitrap MS and included external calibrants with internal standards for a large panel of metabolites [37]. Implementing the internal standardization approach together with the use of high-resolution Orbitrap MS enabled us to perform targeted and non-targeted metabolomics in a single analytical run.

As suggested by the standardization initiative of the Metabolomics Society, pooled MTS extracts were used as quality control samples [43,44]. Combining positive and negative data, absolute concentration values were obtained. Only analytes with technical repeatability obtained from repeated injections of the QC sample below 30% were considered, which resulted in a total number of 58 remaining analytes. The average relative standard deviation (RSD) for the 58 metabolites was 6.5% for the technical replicates and 13% for biological replicates considering each four group (Table S3, pmolMetabolite sheet). The average RSD after the protein content normalization was 15.2% (Table S3, pmolMetabolitePerµgProtein sheet).

**Figure 4:**
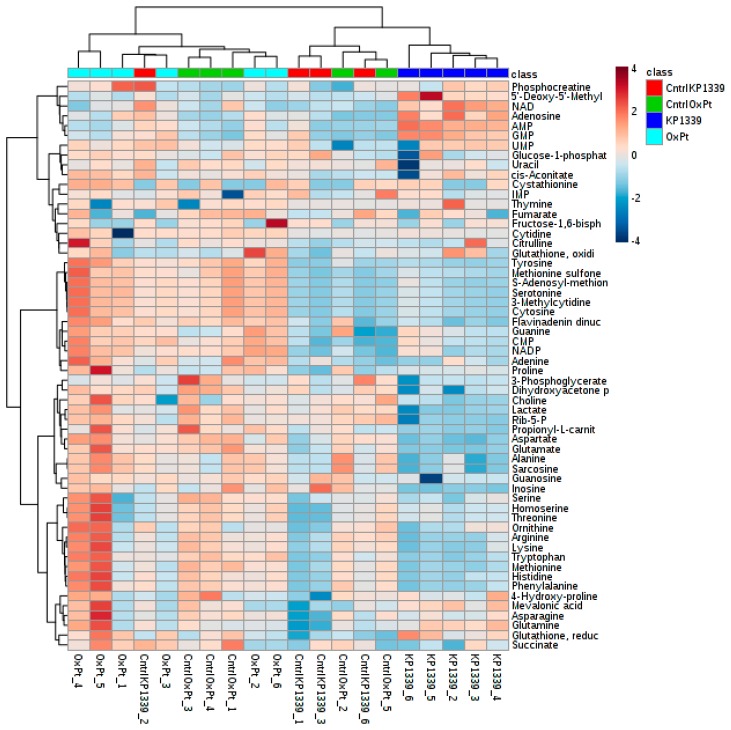
Heatmap displaying absolute amounts of metabolites normalized to total protein content (pmol/µg) of samples from single 3D MTS. Spheroids were treated for 24 h with either 200 µM KP1339, 20 µM oxaliplatin (OxPt), medium (CntrlOxPt) or medium with 0.5% DMSO (CntrlKP1339). Numbers in sample names refer to independent biological replicates. Extraction with cold methanol and internal standardization, measurement by HILIC high-resolution Orbitrap MS.

**Figure A5:**
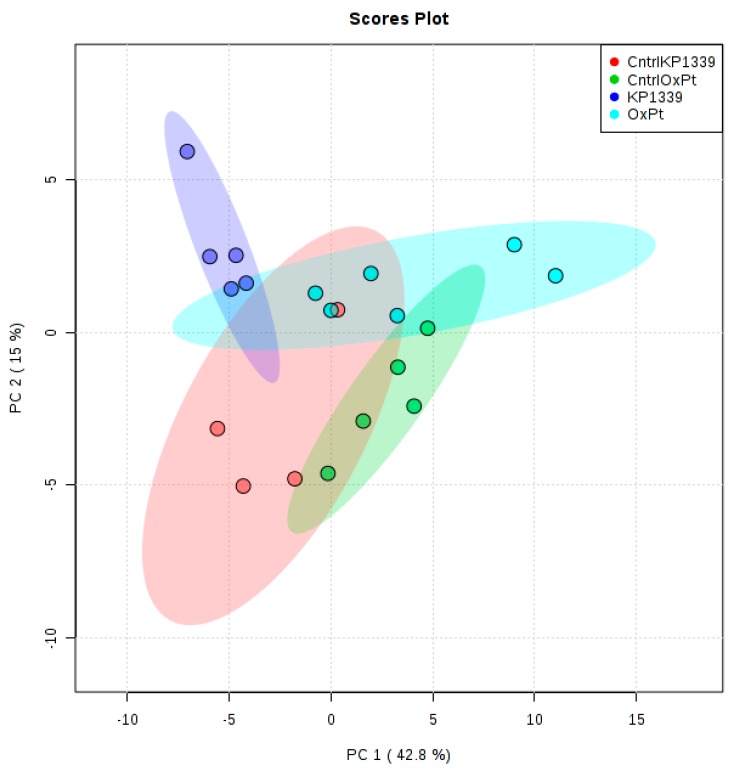
PCA scores plot of absolute amounts of metabolites normalized to protein content of samples from single 3D MTS. Spheroids were treated for 24 h with either 200 µM KP1339, 20 µM Oxaliplatin (OxPt), medium (CntrlOxPt) or medium with 0.5% DMSO (CntrlKP1339). Extraction with cold methanol and internal standardization, measurement by HILIC high-resolution Orbitrap MS.

Unsupervised statistical analysis of the targeted, protein content normalized data revealed that there is group clustering according to the type of drug treatment in the case of KP1339 treatment (PCA plot in Figure A5 and heat map in Figure 4). The KP1339-treated samples showed significant regulation of 19 metabolites (proline, propionyl-L-carnitine, ribulose-5-phosphate/ribose-5-phosphate, adenosine monophosphate, lactate, aspartate, reduced glutathione, guanosine monophosphate, glutamine, inosine, glutamate, N-acetylserine, adenosine, dihydroxyacetone phosphate, asparagine, mevalonic acid, alanine, and sarcosine; Table S3, (“significant_cmpds_KP1339”) compared to six metabolites (adenosine, guanosine monophosphate, cytidine monophosphate, nicotinamide adenine dinucleotide phosphate (oxidized), uridine monophosphate, and uracil; from Table S3 “significant_cmpds_oxaliPt”) in oxaliplatin-treated samples. The stronger metabolic change caused by the ruthenium drug (KP1339) treatment compared to oxaliplatin treatment was further confirmed by hierarchical cluster analysis, where KP1339-treated samples were separated from both controls, which was not the case for oxaliplatin-treated samples (Figure 4). Overall, the fact that we see a stronger effect in the metabolome with KP1339 treatment than with oxaliplatin treatment is not surprising, since in a study with monolayer cell cultures of the same cell line, we observed considerably milder effects with oxaliplatin, as well [37].

**Figure 5:**
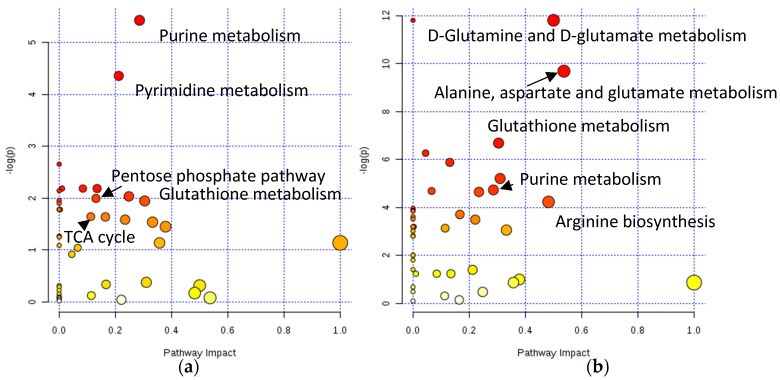
KEGG pathways with MetaboAnalyst affected by oxaliplatin treatment (**a**) and KP1339 treatment (**b**) using pathway enrichment and topology analysis with the MetaboAnalyst pathway analysis module.

Pathway enrichment analysis using targeted data revealed that oxaliplatin exposure affected purine metabolism (GMP and adenosine being most significantly down-regulated; glutamine, IMP, inosine, guanosine, guanine, adenine, and AMP were also affected) and pyrimidine synthesis (UMP and CMP being most significantly down-regulated; but cytidine, uracil, glutamine and thymine were also affected), which is in agreement with the accepted mechanism of action of DNA targeting [45] (Figure 5a, Table S3, “pathways_pathways_oxaliplatin”). These findings were also supported by other significant metabolic shifts involving DNA building blocks. Other retrieved pathways could be related to redox stress such as the pentose phosphate pathway, glutathione metabolism, and nicotinamide metabolism, but also purine metabolism, which is in accordance with the ribosome biogenesis stress only recently proposed as the primary reason for the cytotoxic effect [38]—a hypothesis generated based on transcriptomic analysis. In our work, the measurement of the metabolome provided additional evidence supporting this hypothesis, as the levels of many RNA monomers were significantly altered upon drug exposure (Table S3, “pathways_pathways_oxaliplatin”), and RNA being one of the major component of ribosomes. This was further supported by the fact that the “aminoacyl-tRNA biosynthesis” pathway was also among the affected pathways (see Table S3 for the complete list). Finally, the MetaboAnalyst 4.0 Pathway Analysis module [46] revealed another interesting pathway to be further investigated, namely, the “pantothenate and CoA biosynthesis” pathway (through uracil downregulation). A recent study [29] addressed signature genes for patients responding to oxaliplatin therapy by machine learning. Among the most accurate signature genes for oxaliplatin treatment was PANK3 which encodes for pantothenate kinase, a key regulatory enzyme in the biosynthesis of coenzyme A (CoA). A seminal study correlating transcriptomics and metabolomics for the NCI60 cell line panel showed the involvement of the TCA cycle, pyruvate metabolism, lipoprotein uptake and nucleotide synthesis in platinum sensitivity [47,48]. Again, the metabolomics data of this study support the generated hypothesis as the TCA cycle, purine and pyrimidine metabolism, and pyruvate metabolism were indicated by pathway enrichment analysis.

The hypothesized difference in the mode of action of the two investigated drugs was reflected by the distinct metabolic shifts, which were observed upon exposing MTSs to the candidate ruthenium drug KP1339. There are indications that this drug is a GRP78 inhibitor, an ER stress sensing chaperone [39,49]. It not only induces ER stress and unfolded protein response, but it has also been shown that it exhibits the hallmarks of immunogenic cell death, calreticulin exposure, and ATP secretion among others [42]. Less is known about the metabolic effects of this compound. In this work, we could individuate the impact on biochemical pathways related to redox stress: glutathione metabolism (glutathione, oxidized glutathione and NADP+ are up-, glutamate down-regulated), purine metabolism, pentose phosphate pathway (ribulose 5-phosphate/ribose 5-phosphate down-regulated), which could be explained by the hypothesized reduction of the drug. Redox stress and elevation of ROS protective proteins [42,50] were confirmed by previous proteomics studies. Finally, pathways such as glycerophospholipid metabolism (strong choline down-regulation) and various amino acid metabolism-related pathways were pinpointed (glutamine and glutamate metabolism, alanine, aspartate and glutamate metabolism, arginine biosynthesis). Altered amino acid synthesis is related to the fact that the unfolded protein response induces changes in the expression of genes of amino acid transport and synthesis [51].

## 3. Discussion

In this work, we present a carefully optimized sample preparation workflow for metabolomics experiments in 3D MTS samples. To the best of our knowledge, this is the first study performing extraction optimization (*n* ≥ 4) for single tumor spheroids. The comparison of different extraction strategies, namely ON/BE, OFF/BE, OFF/CM, ON/CM, revealed that cold 80% methanol extraction with a single washing step on the plate was most promising for single tumor spheroid analysis. Using the presented workflow increased throughput and convenience as well as resulted in superior analytical figures of merit including the highest analyte concentration and lowest RSDs observed for 26 metabolites in the pmol to nmol range. The sample preparation strategy is limited to spheroids with diameters >400–500 µm and with maximum diameters of 900–1000 µm due to potential error-prone handling and growth limitation, respectively. Other studies in tumor spheroid analysis were performed using various culturing and extraction protocols such as (1) gelatinous cultivation of spheroids and methanol-water extraction [21], (2) magnetized cells to form 3D cultures and cold acetonitrile, 70% methanol or 80% acetone extraction [22], (3) gel-based ultra-low attachment plates of 20–25 spheroids per sample followed by derivatization and chloroform/methanol extraction [23]. Overall, most –omics mass spectrometry-based studies on tumor spheroid analysis rely on extraction strategies with cold organic solvents such as methanol, ethanol or isopropanol [21,22,23,24]. This is consistent with our tumor spheroid workflow based on non-scaffold based cultivation on ultra-low attachment plates and the optimized cold 80% methanol extraction with a single washing step. The obtained strong linear correlation with the number of (uniform-size) spheroids and relative responses indicates highly repeatable metabolomics readout from single spheroids. The use of ^13^C labeled standards for quantification represents a significant advance compared to label-free quantification approaches as the internal standardization allowed to account for sample losses during sample preparation, storage and measurement. Additionally, ^13^C metabolites represent an interesting normalization strategy for comprehensive non-targeted metabolomics experiments on tumor spheroids. The novel procedure enabled us to probe individual spheroids with excellent biological repeatability (average RSD of 18%) in a considerable throughput. An experiment with a single 96-well plate allows the investigation of up to 60 single spheroids (since wells at the edges are not used). However, high throughput analysis is feasible as there is no theoretical limit in the number of biological replicates or investigated conditions when multiple plates are combined.

The proposed methodology enabled us to investigate different conditions of 3D human cancer models in a parallelized manner comparable to the degree obtained in 2D monolayer cell cultures. The applicability of the method was shown on the example of the metal-based anticancer drugs KP1339 and oxaliplatin. Comparison of the two drugs (*n* ≥ 4) revealed stronger metabolic changes caused by the ruthenium drug (KP1339) treatment compared to oxaliplatin treatment, which is consistent with the literature on monolayer cell cultures [37]. We observed significant metabolic changes with purine and pyrimidine pathways after oxaliplatin treatment, which is in agreement with the accepted mechanism of action of DNA targeting of oxaliplatin [45] and consistent with the proposed induction of ribosome biogenesis stress [38]. Further biological interpretation of the data is beyond the scope of this work.

The presented optimized sample preparation workflow is the ideal starting point for single spheroid metabolomics experiments. A relatively large proportion of clinically tested drugs fail in phase 3 of the trials due to insufficient efficacy or unacceptable toxicity, which means a substantial financial loss [6,52]. Since tumor spheroids are three-dimensional models derived from established human cell lines, it is possible to capture more of the complexity of a tumor compared to standard 2D monolayer cultures [1]. Ultimately, metabolomics studies on tumor spheroids could be integrated into anticancer drug screening, helping to prevent late clinical failure of drug candidates. Future studies will focus on in-depth metabolomics analysis of different anticancer drugs effects using tumor spheroids. Overall, we strongly believe that drug development will benefit significantly from the new discovery tools provided by the unique combination of 3D MTSs and metabolomics.

## 4. Materials and Methods

### 4.1. Cell Culture

#### 4.1.1. Cultivation of Spheroids

The human colon carcinoma cell line HCT116 was kindly provided by Brigitte Marian, Institute of Cancer Research, Department of Medicine I, Medical University of Vienna. HCT116 cells were cultured as adherent monolayers in 75 cm^2^ flasks (StarLab Germany) in McCoy’s 5a medium (Sigma-Aldrich) supplemented with 10% fetal calf serum (FCS) (BioWest) and 4 mM L-glutamine without antibiotics at 37 °C (StarLab) under a humidified atmosphere containing 5% CO_2_. All cell culture media and reagents were obtained from Sigma-Aldrich (Vienna, Austria), and all plastic dishes, plates and flasks were from StarLab (Germany) unless stated otherwise. For spheroid generation, HCT116 cells were harvested from culture flasks by trypsinization, resuspended in their respective supplemented medium, and seeded in 200 µL on ultra-low attachment round-bottom 96-well plates (Nunclon Sphera^TM^, Thermo Fisher Scientific). To avoid effects caused by evaporation, the outermost wells were not used for spheroid production and filled with 200 mL of PBS instead. In the experiment where 1, 5, 10, 15 spheroids were investigated, the spheroids were seeded at a density of 3 × 10^3^ viable cells per well in 200 µL. Plates were incubated at 37 °C with 5% CO_2_ for 96 h to allow spheroid formation. In the experiment where washing procedures and extraction methods were compared, 10 × 10^3^ viable cells were seeded in 200 µL medium and cultivated at 37 °C with 5% CO_2_ for 96 h to allow spheroid formation. In the proof of concept experiment with metallodrugs, 10 × 10^3^ viable cells were seeded in 200 µL medium and cultivated at 37 °C with 5% CO_2_ for 96 h to allow spheroid formation, and then 100 µL medium was aspirated and exchanged for fresh medium. After 192 h (8 days) of total incubation, the diameter of the spheroids was measured, 100 µL of medium was exchanged for the respective treatment solution and 24 h of incubation followed either with the drug or with control medium. KP1339 was first dissolved in DMSO, and stock solutions were prepared in the respective medium with FCS and glutamine supplement. Oxaliplatin was dissolved freshly before the experiments in the supplemented medium only.

#### 4.1.2. Microscopy

An Olympus CKX41 (Olympus, Vienna, Austria) microscope was used to measure the diameter of the spheroids in horizontal and vertical directions with cellF 2.7 imaging software and the average diameter was calculated.

#### 4.1.3. Cell Number Estimation

Spheroids grown from 3 × 10^3^ viable cells for 96 h were transferred from the plate to Eppendorf-tubes as single spheroids. After washing once with PBS, dissociation in 100 µL TrypLE Express (Gibco, Vienna, Austria) followed for 15 min at 37 °C. Samples were thoroughly pipetted to disperse any remaining cell aggregates, and 100 µL colorless McCoy’s 5a medium (Sigma-Aldrich) with 2% FCS were added followed by 0.8 mL Guava ViaCount reagent (Merck/Millipore, Germany). Samples were measured immediately by using a Guava Soft flow cytometer (Merck/Millipore, Darmstadt, Germany).

### 4.2. Methods for Determining the Minimal Number of MTS Required for a Metabolomics Experiment

#### 4.2.1. Sampling 1, 5, 10, 15 Multicellular Tumor Spheroids

3D MTS were seeded with 3 × 10^3^ cells in 200 µL McCoys medium and cultivated for 4 days, reaching a diameter of 560 ± 30 µm. Upon extraction, spheroids were transferred by pipetting to a Petri-dish, where little droplets of PBS had been pre-pipetted. Each spheroid was transferred sequentially into three fresh PBS droplets corresponding to three washing steps. After that, each spheroid was transferred into a conical screw cap vial (Bioquote Limited, York, United Kingdom). The vial was put into liquid nitrogen in order to quench the enzymatic activity and put on wet ice, while more spheroids were collected. As soon as the last spheroid for a given sample was washed and sampled to the vial, it was put on −20 °C. When all the samples were collected, they were stored at −80 °C until boiling ethanol extraction.

#### 4.2.2. Internal Standardization

A fully ^13^C labeled yeast extract of *Pichia pastoris* (2 billion cells) from ISOtopic Solutions e.U., (Vienna, Austria) was reconstituted in 2 mL of water and added in equal amounts to the samples. The final dilution of the internal standard for the measurement was 1:10.

#### 4.2.3. Boiling Ethanol Extraction for 1×-5×-10×-15× MTSs

The protocol was implemented from the works [32,33]. Shortly, 75% ethanol was prepared from ethanol, abs. 100%, HPLC grade (Chem-Lab, Vienna, Austria), H_2_O (Sigma, Vienna, Austria, LC-MS-grade), and pre-heated in a clean glass beaker in a 95 °C water bath. The 50 µL of U^13^C internal standard was added to samples. Once the ethanol–water was boiling in the glass beaker, the hot extraction solvent was added to the conical screw cap vials (Bioquote Limited, York, United Kingdom) to dilute the internal standard amount 1:10 and, subsequently, a 5-min incubation in the 95 °C water bath followed. The vials were closed, vortexed, and centrifuged (14,000 rcf, 4 °C, 5 min). The supernatant was carefully transferred to an MS-vial (Macherey-Nagel, Vienna, Austria), without disturbing the pellet. Extracts were evaporated until dryness. Extracts and resulting pellets were stored at −80 °C until measurement.

#### 4.2.4. Metabolomics Measurement of Extracts 1×-5×-10×-15× MTSs

Samples were reconstituted in 100 µL LC-MS-grade H_2_O and analyzed with reversed phase liquid chromatography coupled to high-resolution Orbitrap mass spectrometry. Waters Acquity HSS T3 (2.1 × 150 mm, 1.8 µm) column was used, mobile phase A was H_2_O (0.1% formic acid), and mobile phase B was 100% methanol. The following gradient was used at a flow rate of 0.3 mL min^−1^ and 40 °C: 0.0–2.0 min 0% B, 2–6 min 0–40% B, 6–8 min 40–100% B, 8–11 min 100% B, and at 11.1 min switch to 0% B, 11.1–15 min 0% B. The injection volume was 10 µL. The Vanquish Duo UHPLC-system (Thermo Fisher Scientific) was used.

High-resolution mass spectrometry was done with a high field Thermo Scientific™ Q Exactive HF™ quadrupole-Orbitrap mass spectrometer equipped with an electrospray source. The ESI source parameters were the following: sheath gas 40, auxiliary gas 3, spray voltage 2.8 kV in negative and 3.5 kV in the positive mode, capillary temperature 280 °C, S-Lens RF level 30 and auxiliary gas heater 320 °C. Spectral data were acquired in profile mode. Resolution = 60.000, mass range = 60–900 m/z, AGC target 10^6^, both in positive and negative polarities. Quantification was done through the areas of extracted ion chromatograms of [M+H]^+^ and [M-H]^−^ with 5 ppm mass tolerance on the U^12^C to U^13^C ratio.

#### 4.2.5. Acidic Hydrolysis of the Pellet and Amino Acid Analysis

Acidic hydrolysis of the pellet resulting from the boiling ethanol extraction of samples of 1, 5, 10, and 15 spheroids pooled was based on a procedure described in detail elsewhere [53,54]. In short, 1 mL 6 M hydrochloric acid (Fluka, TraceSELECT from ≥37%) was added to the pellets, already in the conical screw cap vials (Bioquote Limited, York, United Kingdom); screw caps were tightly closed and the samples were hydrolyzed at 100 °C for 24 h. The hydrolyzed samples were evaporated to dryness and stored at −80 °C until analysis. Procedural blanks were also prepared to investigate possible background. Upon measurement, samples were reconstituted in 1 mL H_2_O. A 1-to-10 dilution was done by taking 50 µL from the sample, adding 50 µL ISTD and 400 µL acetonitrile. NIST SRM 2389a amino acid standard (Sigma-Aldrich) was used to prepare an external calibration with internal standard and achieve absolute concentrations. The eight amino acids were subject to quantitative extraction and measurement according to [36] (alanine, arginine, glycine, histidine, lysine, phenylalanine, proline, tyrosine) were measured with a method described in detail elsewhere [35]. Shortly, the Agilent Infinity LC-System coupled to an Agilent 6490 triple quadrupole mass spectrometer was used with a HILIC separation, Waters Acquity BEH Amide (1.7 µm, 100 × 0.786 mm) column with 10 mM ammonium formate (pH 3.25) as eluent A and 80% acetonitrile 20% 10 mM ammonium formate (pH 3.25) as eluent B. Multiple reaction monitoring transitions were acquired in positive polarity.

### 4.3. Methods for Comparison Boiling Ethanol (BE) and Cold Methanol (CM) Extraction and Washing Procedures (OFF vs. ON) of 3D MTS

For this experiment, 3D MTS were grown as described above from 10 × 10^3^ cells for 96 h.

#### 4.3.1. Transfer of Spheroids

Sampling and transfer of spheroids were carried out with a 200 µL (Eppendorf, Vienna, Austria) pipette. The tip of the pipette tip was cut with a scissor (washed before in methanol:H_2_O 50:50) so that the opening was slightly enlarged. The spheroid was taken up with the surrounding solution by the suction generated by the pipette and transferred from the plate into an Eppendorf tube.

#### 4.3.2. “BE-OFF” Sample Preparation

The spheroids were transferred from the plate into conical screw cap vials (Bioquote Limited, York, United Kingdom), where they were washed three times with phosphate-buffered saline (PBS, Sigma-Aldrich, Vienna, Austria), quenched immediately with liquid nitrogen and stored at −80 °C until extraction. Upon extraction, 20 µL U^13^C internal standard was added as well as 180 µL 75% ethanol and put on 95 °C in the water bath for 5 min. After 5 min of sonication, samples were vortexed and centrifuged (14,000 rcf, 5 min, 4 °C) and supernatants transferred into MS-vials. Samples were measured directly without evaporation.

#### 4.3.3. “CM-OFF” Sample Preparation

The spheroids were transferred from the plate into an Eppendorf tube, where they were washed three times with PBS and quenched immediately in liquid nitrogen and stored at −80 °C until extraction. Upon extraction, 20 µL U^13^C internal standard was added as well as 180 µL cold 80% methanol (−20 °C). After 5 min of sonication, samples were vortexed and centrifuged (14,000 rcf, 5 min, 4 °C) and supernatants transferred into MS-vials. Samples were measured directly without evaporation.

#### 4.3.4. “BE-ON” Sample Preparation

The medium of spheroids was carefully aspirated by a multichannel pipette, while spheroids remained in the wells. Subsequently, the spheroids were washed with 200 µL PBS and quenched by liquid nitrogen. The spheroids still in the wells were stored at −80 °C until extraction. Plates were kept on ice during extraction. Upon extraction, 20 µL U^13^C internal standard was added to the well, then 80 µL 75% ethanol (room temperature, not hot yet) was added; in this amount, the spheroid was transferred to conical screw cap vials (Bioquote Limited, York, United Kingdom). One hundred µL more 75% ethanol was added to wash the well and transferred to the conical screw cap vials (Bioquote Limited, York, United Kingdom). Samples were kept on ice until the extraction of all samples was completed. Samples were vortexed and put on 95 °C in the water bath for 5 min, then 5 min of sonication followed. Finally, samples were centrifuged (14,000 rcf, 5 min, 4 °C), and supernatants were transferred into MS-vials. Samples were measured directly without evaporation.

#### 4.3.5. “CM-ON” Sample Preparation

The medium of spheroids was carefully aspirated by a multichannel pipette, while spheroids remained in the wells. Subsequently, the spheroids were washed with 200 µL PBS and quenched by liquid nitrogen. The spheroids still in the wells were stored at −80 °C until extraction. Plates were kept on ice during extraction. Upon extraction, 20 µL of the U^13^C internal standard was added, then 80 µL cold 80% methanol was added (−20 °C); in this amount, the spheroid was transferred to an Eppendorf-tube and 100 µL more cold 80% methanol was added to wash the well and transferred to the Eppendorf tube. Samples were kept on ice until the extraction of all samples was completed. After 5 min of sonication, samples were vortexed and centrifuged (14,000 rcf, 5 min, 4 °C), and the supernatants were transferred into MS-vials. Samples were measured directly without evaporation.

#### 4.3.6. LC-MS Method Applied for Proof of Principle Experiment with Metallodrugs

The metabolite standards were purchased from Sigma-Aldrich or Fluka (Vienna, Austria) except for malic acid which was purchased from Merck (Vienna, Austria). The metabolomics experiment also included an external calibration with a calibration mix of 133 substances and internal standardization with U^13^C-labeled yeast extract. Within every 10 injections, a blank was injected, as well as a pooled QC from extracts (all four groups represented in each: 200 µM KP1339-treated, 20 µM oxaliplatin-treated, control, control with 0.5% dimethylsulfoxid (DMSO)). We used high resolution Orbitrap MS coupled to Vanquish UPLC and with a HILIC separation, as described elsewhere [37]. MS-data were acquired with positive–negative switching and the extracted ion chromatograms were evaluated with Thermo Trace Finder, with the help of external calibration and the internal standard, absolute amounts were calculated in pmol. The normalization with total protein content resulted in pmol metabolite per µg protein.

#### 4.3.7. Total Protein Content Determination

The protein concentration was assessed from the precipitate dissolved in 200 µL 0.05 M NaOH. For that purpose, the commercially available micro BCA protein assay kit (Thermo Fisher Scientific, Pierce Biotechnology, Rockford, IL, USA) was employed, according to the manufacturer’s instructions.

### 4.4. Data Analysis

#### 4.4.1. Targeted Metabolomics Data Treatment and Normalization

Targeted data evaluation of high-resolution mass spectrometry data of metabolites was performed in Thermo Trace Finder 4.1, while for the triple quadrupole data of amino acids hydrolyzed from pellet Agilent MassHunter was used. ^13^C internal standardization using external calibration was employed for metabolites. All calibration curves were linear.

The dataset with absolute metabolite amounts and total protein contents was exported to Python, where the normalization with protein content was carried out, as well as filtering the dataset based on the performance of pooled quality control samples. (Missing values > 50%, relative standard deviation above 30%).

#### 4.4.2. Exploratory Data Analysis

The MetaboAnalyst 4.0 Statistical Analysis module was used for further exploratory data analysis of dataset with absolute metabolite amounts normalized to total protein content. The default missing values imputation was used with a small number; autoscaling was applied before multivariate analysis.

#### 4.4.3. Pathway Analysis

The MetaboAnalyst 4.0 [46] Pathway Analysis module was used, which combines pathway enrichment analysis and pathway topology analysis to identify the most relevant pathways involved in the conditions under study. As data input, the whole dataset with protein content normalized concentrations was used. Normalized concentrations were autoscaled; missing values imputed with a small number. The following parameters were used: pathway enrichment with “global test”, pathway topology analysis with “relative-betweenness centrality”, pathway library was homo sapiens KEGG, KEGG version Oct 2019.

## Figures and Tables

**Table 1:** Average relative standard deviations based on absolute concentrations of 26 analytes in four investigated sample preparation strategies for single multicellular tumor spheroids. The four different sample preparation methods involved two washing procedures (“ON”: washing the multicellular tumor spheroid once with PBS on the 96-well plate vs. “OFF” washing three times in an Eppendorf tube after transfer from cultivation well) and two alternative extractions are combined (boiling ethanol “BE” vs. cold methanol extraction “CM”). OFF/BE (*N* = 6), ON/BE (*N* = 4), OFF/CM (*N* = 5), ON/CM (*N* = 7) One pooled sample from independent OFF/CM and ON/CM samples, QC was a mixture of one OFF/CM and ON/CM sample and was measured as quality control throughout the measurement (*N* = 4). Measurement was performed with liquid chromatography (HILIC separation) high-resolution Orbitrap mass spectrometry. All extractions utilized U^13^C internal standardization.

Sample Preparation	Average RSD [%]
OFF/BE	34%
ON/BE	19%
OFF/CM	24%
ON/CM	18%
QC	7%

**Figure A1:**
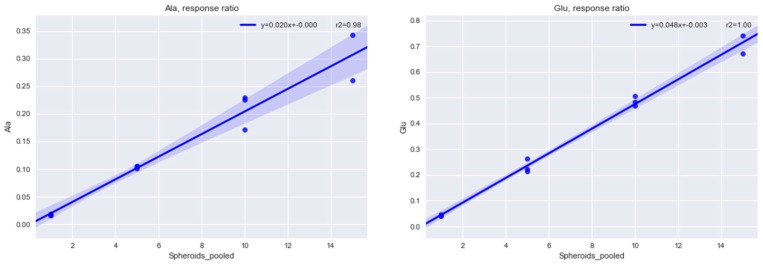
Relative responses (^12^C/^13^C) of alanine and glutamate as selected examples from a single (*N* = 3), 5 (*N* = 3), 10 (*N* = 3), 15 (*N* = 2) multicellular tumor spheroids. Sample preparation applied boiling ethanol extraction with U^13^C internal standardization and LC high-resolution mass spectrometry. Investigated metabolites showed high linear correlation.

## Supplementary Materials

The following are available online at https://www.mdpi.com/2218-1989/9/12/304/s1, Table S1: 1x_5x_10x_15x_MTS. Table S2: washing extractions comparison, Table S3: proof Experiment Metallodrugs.

## Abbreviations

Abbreviations of metabolites and their explanation used in this work.

Abbreviation	Explanation
5AMP, AMP	adenosine monophosphate
5CMP, CMP	cytidine monophosphate
5GMP, UMP	guanosine monophosphate
5UMP	uridine monophosphate
AAA	alpha aminoadipic acid
Ade	adenine
ADP	adenosine diphosphate
Ala	alanine
Arg	arginine
Asin	adenosine
Asn	aspragine
Asp	aspartic acid
DHAP	dihydroxyacetone phosphate
Gln	glutamine
Glu	glutamate
Gly	glycine
GMP	guanosine monophosphate
GSH	glutathione (reduced)
Gsin	guanosine
GSSG	Glutathione (oxidized)
His	histidine
H-Ser	homoserine
IMP	inosine monophosphate
Isin	inosine
K-Ile	ketoisoleucine
Lys	lysine
Met	methionine
NAD+	nicotinamide adenine dinucleotide (oxidized)
NADP+	nicotinamide adenine dinucleotide phosphate (oxidized)
OAS	O-acetylserine
Phe	phenylalanine
Pro	proline
Pyr	pyruvate
Rib-5-P	ribose 5-phosphate/ribulose 5-phosphate
Ser	serine
Thr	threonine
Trp	tryptophan
Tyr	tyrosine

## Appendix A

Table S1. Summary of the experiment which had the aim of determining the minimal number of MTS required for a metabolomics experiment. Relative responses of 1, 5, 10 and 15 MTS from the boiling ethanol extracts. Absolute amounts of amino acids [nmol] from hydrolysed pellets. Cell numbers and viability. Parameters of the normalized linear regressions.

Table S2. Summary of the experiment comparing the different sample preparation strategies with three washing steps vs. single washing on the plate and boiling ethanol vs. cold methanol extraction. Summary of the absolute amounts of selected metabolites. Individual absolute amounts in samples.

Table S3. Summary of the proof of principle experiment with metallodrugs. Absolute amounts of metabolites in samples and RSDs. Absolute amounts of metabolites normalized to total protein content and RSDs. Absolute amounts of metabolites normalized to total protein content with missing value imputation. Absolute amounts of metabolites normalized to total protein content with missing value imputation and autoscaling. Total protein content in the samples. KEGG pathways affected by Oxaliplatin treatment. KEGG pathways affected by KP1339 treatment. Significant compounds (*p* < 0.05) based on oxaliplatin treatment. Significant compounds (*p* < 0.05) based on KP1339 treatment.
